# Cascade-responsive HA-decorated liposome/mesoporous silica nanoplatform co-delivering BMF-219 and 5-FU enhances gastric cancer chemotherapy through Menin- and p53-associated apoptotic signaling

**DOI:** 10.1186/s13046-026-03784-6

**Published:** 2026-07-15

**Authors:** Shanhu Wang, Dongsheng Li, Fanshuo Zeng, Runchuan Wang, Chhingher Ly, Jinxun Jiang, Zhen Wang

**Affiliations:** 1 Department of Gastrointestinal and Glandular Surgery, The First Affiliated Hospital of Guangxi Medical University, Guangxi Zhuang Autonomous Region 530021 Nanning, China; 2 Guangxi Key Laboratory of Enhanced Recovery after Surgery for Gastrointestinal Cancer, Nanning, Guangxi Zhuang Autonomous Region 530021 China

**Keywords:** Gastric cancer, Chemotherapy resistance, Menin inhibitor, p53 signaling pathway, Multistage responsive nanoplatform

## Abstract

**Background:**

Our preliminary study found that Men1 is an oncogene and a potential therapeutic target in gastric cancer (GC). Chemotherapy remains a common therapeutic regimen for GC, but is frequently limited by multidrug resistance and poor intratumoral coordination of combination drugs. Targeting Menin may enhance chemotherapy sensitivity, but efficient co-delivery of Menin inhibitors and chemotherapeutics is hindered by the profound spatiotemporal pharmacokinetic decoupling of the drugs in vivo.

**Methods:**

We first evaluated the synergistic antitumor effect and Menin/p53-associated mechanism of BMF-219 combined with 5-fluorouracil (5-FU) in gastric cancer models. We then engineered a hierarchically assembled, cascade-responsive hyaluronic acid (HA)-decorated liposome/mesoporous silica nanoplatform (HLM@BF) for coordinated co-delivery of BMF-219 and 5-FU. The system features a glutathione (GSH)-degradable disulfide-bridged mesoporous silica core, cloaked with a pH-sensitive lipid bilayer and decorated with HA for targeting. The synergistic efficacy and biological mechanism were evaluated through bioinformatic analysis, RNA sequencing (RNA-seq), in vitro assays, and in vivo experiments.

**Results:**

BMF-219 sensitized GC cells to 5-FU-induced apoptosis through modulation of Menin- and p53-associated apoptotic signaling. HLM@BF was constructed successfully, and showed colloidal stability in serum-containing medium, pH/GSH-responsive co-delivery, enhanced CD44-associated uptake, endo/lysosomal redistribution or escape, increased tumor accumulation, and suppressed xenograft growth with favorable biosafety.

**Conclusions:**

HLM@BF integrates Menin inhibition with 5-FU chemotherapy and enhances antitumor efficacy through Menin- and p53-associated apoptotic signaling, providing a targeted nanomedicine strategy for gastric cancer.

**Supplementary Information:**

The online version contains supplementary material available at https://doi.org/10.1186/s13046-026-03784-6.

## Introduction

Gastric cancer (GC) remains one of the most prevalent and lethal malignancies worldwide, presenting a formidable challenge to human health [1]. Although 5-fluorouracil (5-FU)-based combination chemotherapy is a standard first-line treatment for advanced GC, its clinical efficacy is hampered by the rapid emergence of multidrug resistance (MDR) and severe systemic toxicities [2]. The underlying cause of this therapeutic bottleneck often stems from inactivation of intrinsic tumor suppressor pathways and subsequent evasion of apoptosis [3, 4]. Specifically, the p53 signaling pathway orchestrates cell-cycle arrest and apoptosis in response to 5-FU-induced DNA damage. The cytotoxic efficacy of 5-FU is highly dependent on the functional integrity of p53 [5]. Thus, suppression or mutational inactivation of the p53 pathway is a major driver of both primary and acquired 5-FU resistance in cancer [6]. Therefore, identifying novel targets to reverse this resistance and developing effective drug-delivery systems are imperative to overcome the current impasse in GC treatment.

Emerging evidence highlights the critical role of epigenetic dysregulation in driving tumor chemoresistance [7]. In this context, Menin, a multifunctional transcriptional regulator encoded by the Men1 gene, has attracted significant attention. While classically recognized as a tumor suppressor in endocrine tissues, Menin exhibits a paradoxical, context-dependent oncogenic role in certain malignancies [8]. For instance, in hepatocellular carcinoma, Menin promotes hepatocarcinogenesis and epigenetically upregulates Yap1 transcription [9], while pharmacologic inhibition of the Menin-MLL interaction represses PEG10 and blocks HCC progression [10]. Furthermore, our preliminary study in GC has shown that cancer-associated fibroblasts (CAFs) express high levels of Menin and deliver it to GC cells via exosomes. This CAFs-exosomes-Menin axis activates the HSPA6/JNK/JunD signaling pathway to induce epithelial–mesenchymal transition (EMT), highlighting its critical role in GC progression within the tumor microenvironment [11].

Moreover, the interaction between Menin and p53 has garnered extensive attention across various tumor types. In pancreatic neuroendocrine tumors, Men1 co-regulates INSM1 expression and localization alongside p53 and Notch; Men1 loss leads to nuclear INSM1 accumulation and hyperproliferation [12]. In breast cancer, the Menin–Dot1L chromatin complex regulates genes within the p53 signaling pathway, and targeting this complex inhibits the proliferation of endocrine therapy-resistant cells [13]. These findings underscore that the function of the Menin-p53 axis is highly tumor-type dependent and can exert either oncogenic or tumor-suppressive effects via epigenetic reprogramming. Consequently, we hypothesize that reducing Menin abundance or inhibiting Menin function in GC could relieve suppression of p53-associated apoptotic signaling and resensitize GC cells to 5-FU.

Menin-MLL inhibitors have been explored in other disease contexts [14], while BMF-219 (icovamenib) is a covalent Menin inhibitor under clinical investigation, with early clinical evidence reported in NUP98 fusion-positive acute myeloid leukemia [15]. More broadly, Menin inhibitors are increasingly recognized as a therapeutic class in hematologic malignancies [16]. In recent years, combining 5-FU with pathway-targeted modulators has emerged as a promising strategy to improve antitumor efficacy and counter chemoresistance [17]. Guided by this paradigm, the co-administration of BMF-219 and 5-FU holds strong clinical potential. However, translating this drug combination into clinical application is hindered by significant pharmacokinetic mismatches. The disparate physicochemical properties of the two agents (hydrophilic 5-FU and highly hydrophobic BMF-219) can lead to spatiotemporal decoupling in vivo, preventing them from reaching the tumor site at optimal synergistic ratios [18, 19]. Furthermore, nanocarrier formulation can substantially alter systemic exposure and tolerability of cytotoxic agents, as illustrated by clinical pharmacokinetic studies of polymeric micelle-formulated paclitaxel [20].

Nanomedicine offers a practical strategy to overcome the pharmacokinetic mismatch between combination drugs with distinct physicochemical properties. For the BMF-219/5-FU (BF) combination, the highly hydrophobic nature of BMF-219 and the hydrophilic nature of 5-FU may lead to asynchronous tumor delivery, premature systemic exposure, and suboptimal intratumoral drug ratios. Therefore, a co-delivery system capable of coordinating drug loading, tumor accumulation, intracellular release, and mechanism-linked therapeutic action is needed.

To address this challenge, we engineered a hyaluronic acid (HA)-decorated, pH-sensitive liposome/mesoporous silica nanoplatform (HLM@BF) for coordinated delivery of BMF-219 and 5-FU. The intended distinctiveness of HLM@BF lies in its formulation-function-mechanism integration for this specific drug pair: HA decoration supports CD44-associated tumor uptake, the pH-sensitive lipid layer facilitates endo/lysosomal destabilization, and the glutathione (GSH)-responsive disulfide-bridged silica core promotes intracellular payload release. In this study, we first evaluated the synergistic antitumor effect and Menin/p53-associated apoptotic mechanism of BMF-219 combined with 5-FU, and then assessed whether HLM@BF could enhance this combination through cascade-responsive co-delivery in vitro and in vivo.

## Materials and methods

### Cell culture and antibodies

The human gastric cancer cell lines SNU-216 (RRID: CVCL_3946) and MKN-28 (RRID: CVCL_1416) were obtained from Wuhan Pricella Biotechnology Co., Ltd. (Wuhan, China). All cell lines were authenticated using short tandem repeat (STR) profiling and were routinely confirmed to be negative for mycoplasma contamination. Cells were cultured in RPMI-1640 medium (Gibco, USA) supplemented with 10% fetal bovine serum (FBS; Biological Industries, Israel), 50 µg/mL streptomycin, and 50 U/mL penicillin (Solarbio, Beijing, China). The cell cultures were maintained in a humidified incubator at 37 °C with 5% CO₂.

Primary antibodies utilized were as follows: anti-Menin (Bethyl, Cat# A300-105A, RRID: AB_2143306; 1:8000 for Western blotting (WB), 1:500 for immunohistochemistry (IHC)), anti-p53 (Proteintech, Cat# 60283-2-Ig; 1:1000 for WB, 1:200 for IHC), anti-phospho-p53 (Ser15) (Proteintech, Cat# 28961-1-AP; 1:1000 for WB, 1:200 for IHC), anti-Bax (ZenBio, Cat# R380709; 1:1000 for WB, 1:100 for IHC), anti-Bcl-2 (Huabio, Cat# ET1702-53; 1:5000 for WB, 1:500 for IHC), anti-Ubiquitin (Proteintech, Cat# 10201-2-AP; used for immunoprecipitation-Western blotting (IP-WB) according to the manufacturer’s protocol), and anti-ACTB (Proteintech, Cat# 20536-1-AP; 1:10000 for WB). Horseradish peroxidase (HRP)-conjugated goat anti-rabbit IgG and goat anti-mouse IgG secondary antibodies were supplied by Proteintech (Wuhan, China).

### Cell viability and synergism analysis

Cell viability was assessed using the Cell Counting Kit-8 (CCK-8) assay. Briefly, SNU-216 and MKN-28 cells were seeded into 96-well plates (5 × 10³ cells/well) and cultured overnight. Cells were then treated for 48 h with concentration gradients of free 5-FU, free BMF-219, or their combination (BF), as specified in the corresponding figure panels. After treatment, CCK-8 reagent (Solarbio, Beijing, China) was added, and cells were incubated at 37 °C for 1 h. Absorbance at 450 nm was measured using a multimode microplate reader (Tecan, Switzerland). The half-maximal inhibitory concentration (IC50) values were calculated using GraphPad Prism 9.5. To evaluate synergistic interactions, dose-response matrix data were analyzed using the SynergyFinder web platform (https://www.synergyfinderplus.org) [21], and synergy scores were computed using the Highest Single Agent (HSA) reference model [22].

### Immunoprecipitation (IP) and ubiquitination assay

To evaluate the ubiquitination status of Menin, SNU-216 cells were treated with dimethyl sulfoxide (DMSO) or BMF-219 for 24 h, with or without the addition of the proteasome inhibitor MG-132 (10 µM) during the final 6 h of incubation. Total cell lysates were harvested using an IP lysis buffer supplemented with protease and phosphatase inhibitors. Equivalent amounts of protein lysates were incubated with an anti-Menin antibody or normal IgG (as a negative control) overnight at 4 °C, followed by the addition of Protein A/G magnetic beads for 2 h. The immunoprecipitated complexes were eluted in sodium dodecyl sulfate (SDS) loading buffer and subjected to Western blot analysis utilizing an anti-Ubiquitin antibody to detect polyubiquitinated Menin species.

### EdU proliferation assay

Following the designated treatments, cellular proliferation was evaluated using a BeyoClick™ EdU-555 Cell Proliferation Assay Kit (Beyotime, Shanghai, China). Cells were incubated with EdU for 2 h, followed by fixation, permeabilization, and Apollo staining according to the manufacturer’s protocol. Nuclei were counterstained with 4’,6-diamidino-2-phenylindole (DAPI). Fluorescence images were acquired under identical settings within each experiment using an inverted fluorescence microscope (Nikon, Japan). The proliferation rate was quantified as the ratio of EdU-positive cells (red) to DAPI-positive cells (blue) (*n* = 3).

### Invasion and migration assays

Following the designated treatments, cellular invasion and migration capacities were assessed using 24-well Transwell chambers (8.0 μm pore size; Corning, USA). For the invasion assay, the upper chambers were pre-coated with Matrigel (200 µg/mL; Corning/BD Biosciences, USA; Cat# 356234). Cells suspended in serum-free medium (2 × 10⁵ cells/mL, 100 µL) were seeded into the apical chamber, while medium supplemented with 10% FBS was added to the basolateral chamber as a chemoattractant. After 24 h, non-migrated cells on the apical surface were gently removed with cotton swabs. Migrated or invaded cells on the basal surface were fixed with 95% ethanol, stained with 0.1% crystal violet, imaged using an inverted optical microscope (Olympus, Japan), and counted quantitatively. Migration assays followed the same protocol without Matrigel coating (*n* = 3).

### Cell apoptosis analysis

Following 48 h of the designated therapeutic interventions, cells were harvested, washed twice with cold phosphate-buffered saline (PBS), and resuspended in binding buffer. Apoptosis was assessed using an Annexin V-FITC/PI Apoptosis Detection Kit (MultiSciences, Hangzhou, China) according to the manufacturer’s instructions. Samples were acquired on a flow cytometer (BD FACSCanto; BD Biosciences, USA). The total apoptotic rate, including Q2 late apoptotic and Q3 early apoptotic populations, was analyzed using FlowJo software (v10.8, BD Biosciences, Ashland, OR, USA) with the same gating strategy across groups.

### RNA extraction, transcriptomic sequencing (RNA-seq), and bioinformatics

Bioinformatic screening was performed to contextualize the relationship among 5-FU response, TP53 signaling, and Men1 expression. Fluorouracil-associated genes were retrieved from the Comparative Toxicogenomics Database (CTD; https://ctdbase.org), and the top interacting genes were visualized. The correlation between TP53 and Men1 expression in GC was analyzed using Gene Expression Profiling Interactive Analysis 2 (GEPIA2; http://gepia2.cancer-pku.cn) based on The Cancer Genome Atlas (TCGA) data. Men1 expression in GC tumor and normal tissues was further evaluated using The Cancer Genome Atlas stomach adenocarcinoma cohort (TCGA-STAD) data through the UCSC Xena platform (https://xena.ucsc.edu).

For the nanoplatform-related transcriptomic analysis, total RNA was extracted from HLM@BF-treated SNU-216 cells and normally cultured SNU-216 controls, with three biological replicates per group. HLM@BF treatment was performed under the same condition used for the corresponding in vitro mechanistic assays. RNA-seq library construction and sequencing were performed by Sangon Biotech (Shanghai, China) on the Illumina NovaSeq 6000 platform. Raw reads were quality-controlled, aligned to the reference genome, and quantified at the gene level before differential expression analysis. Differentially expressed genes (DEGs) were identified using DESeq2 with cutoff criteria of |log2FoldChange| > 0.585 and adjusted *P* < 0.05. Kyoto Encyclopedia of Genes and Genomes (KEGG) pathway enrichment analysis was performed using clusterProfiler.

For Menin-related contextual analysis, our previously generated in-house RNA-seq dataset from Men1-overexpressing SNU-216 cells and corresponding vector controls was analyzed. The transcriptomic dataset GSE139331, derived from 5-FU-treated gastric cancer cell lines, was retrieved from the Gene Expression Omnibus (GEO) database as an external public dataset for 5-FU-associated pathway analysis.

### Lentiviral transfection and rescue cell line construction

For gain-of-function rescue experiments, lentiviral vectors encoding the Men1 overexpression sequence (Men1-OE) and empty negative control vectors were engineered by Hanbio Biotechnology (Shanghai, China). GC cells were transduced with lentiviral particles at a multiplicity of infection (MOI) of 100. At 48 h post-transduction, stable transductants were selected using medium containing 4 µg/mL puromycin for 2 weeks and were subsequently maintained in 1 µg/mL puromycin. Men1 overexpression was verified by qRT-PCR and Western blot analysis.

### Evaluation of CD44 mRNA expression, surface CD44 detection, and knockdown efficiency

Basal CD44 mRNA expression in SNU-216 and MKN-28 cells was assessed by qRT-PCR as described below. Surface CD44 expression was then evaluated in SNU-216 cells by flow cytometry. Briefly, SNU-216 cells were harvested and incubated with a PE-conjugated anti-human CD44 monoclonal antibody or the corresponding isotype control at 4 °C for 30 min in the dark. Surface CD44 expression was analyzed using a flow cytometer. For the targeting mechanism study, SNU-216 cells were transfected with small interfering RNA (siRNA) targeting CD44 (siCD44) or negative-control siRNA using Lipofectamine 3000 according to the manufacturer’s protocol. At 48 h post-transfection, reduced CD44 surface expression was confirmed using the same flow-cytometric protocol before nanoparticle uptake assays.

### Quantitative real-time PCR (qRT-PCR)

To evaluate gene expression levels, total RNA was isolated using the NucleoZOL RNA Isolation Kit (MACHEREY-NAGEL, Düren, Germany). Reverse transcription of 1 µg total RNA into cDNA was performed using HiScript III RT SuperMix (+ gDNA wiper; Vazyme, Nanjing, China). qRT-PCR was conducted on a 7500 Real-Time PCR System (Applied Biosystems, USA) using ChamQ SYBR qPCR Master Mix (Vazyme, Nanjing, China). The cycling conditions were 95°C for 30 s, followed by 40 cycles of 95°C for 10 s and 60°C for 30 s. Primer sequences synthesized by Sangon Biotech (Shanghai) Co., Ltd. (Shanghai, China) were as follows: CD44 forward, 5’-ACTGCTTATGAAGGAAACTGGAACC-3’ and reverse, 5’-TGCCTGGATTGTGCTTGTAGAATG-3’; Men1 forward, 5’-CCCATGTACTTACAAGCCGATAT-3’ and reverse, 5’-CCCCATTTTCGTAAACACACTC-3’; GAPDH forward, 5’-GTGGCCGAGGACTTTGATTG-3’ and reverse, 5’-CCTGTAACAACGCATCTCATATT-3’. Relative mRNA expression was calculated using the 2^-ΔΔCt method, normalized to endogenous GAPDH.

### Preparation and physicochemical characterization of nanoparticles

#### Reagents and instruments

All reagents were acquired from commercial vendors and used without further purification. 5-Fluorouracil (5-FU; CAS: 51-21-8) was purchased from MedChemExpress (MCE, NJ, USA), and the Menin inhibitor BMF-219 (CAS: 2448172-22-1) was obtained from GlpBio (Montclair, CA, USA). Fluorescent dye Cy5.5, lipids [1,2-dioleoyl-sn-glycero-3-phosphoethanolamine (DOPE), cholesteryl hemisuccinate (CHEMS), DSPE-PEG2000, and DSPE-PEG2000-HA; HA molecular weight, 50 kDa], and silica precursors (CTAT, TEA, TEOS, and BTESPD) were supplied by Xi’an Ruixi Biological Technology Co., Ltd. (Xi’an, China). Morphological and physicochemical characterizations were conducted using transmission electron microscopy (TEM; FEI TF20, USA), dynamic light scattering (DLS; NanoBrook 90plus PALS; Brookhaven, USA), Fourier transform infrared spectroscopy (FTIR; Shimadzu IRspirit-X, Japan), and high-performance liquid chromatography (HPLC; Elite T3200, China). Liposome extrusion was performed using a Mini-extruder (Avanti Polar Lipids, AL, USA) equipped with polycarbonate membranes (Whatman, USA).

#### Synthesis of disulfide-bridged mesoporous silica (M)

The disulfide-bridged mesoporous silica core (M) was synthesized via a co-condensation method. Briefly, CTAT (0.2 g) and TEA (0.1 mL) were dissolved in ultrapure water (20 mL) at 80 °C. TEOS (1 mL) and BTESPD (0.5 mL) were then rapidly injected, and the mixture was stirred at 80 °C for 2 h. The resulting white precipitate was collected by centrifugation (12,000 rpm, 10 min, 4 °C) and washed three times with ultrapure water. To remove the CTAT template, the precipitate was refluxed in acidic ethanol (HCl: ethanol = 1:10, v/v) at 70 °C overnight. The purified product (M) was collected, washed with ethanol and water, and stored at 4 °C.

#### Preparation of BF-loaded disulfide-bridged mesoporous silica nanoparticles (M@BF)

M (250 mg) was incubated with 5-FU (20 mg), BMF-219 (20 mg), and Cy5.5 (15 mg) under continuous stirring overnight at room temperature in the dark to allow adsorption and encapsulation of the payloads within the mesoporous channels. After loading, free or weakly associated payloads were removed by centrifugation (13,000 rpm, 15 min, 4 °C) and washing, and the actual drug loading was quantified by HPLC.

#### Preparation of pH-sensitive and HA-modified liposomes

pH-sensitive liposomes (Lipo) were prepared using the thin-film hydration technique. DOPE (45 mg), DSPE-PEG2000 (2.5 mg), and CHEMS (2.5 mg) were dissolved in 2 mL of chloroform. The organic solvent was evaporated under reduced pressure using a rotary evaporator (37 °C, 30 min) to yield a homogeneous lipid film. Hydration was performed with 3 mL of ultrapure water at 37 °C for 1 h, followed by probe sonication in an ice bath (300 W, 5 min). The resulting suspension was extruded 11 times through a 200 nm polycarbonate membrane to obtain uniform vesicles. For HA-modified liposomes (HA-Lipo), DSPE-PEG2000 was replaced with DSPE-PEG2000-HA (2.5 mg; HA molecular weight, 50 kDa) under the same preparation conditions. Because HA was introduced through the amphiphilic DSPE-PEG-HA component within the liposomal layer, the formulation is described as HA-decorated rather than post-synthetically HA-grafted.

#### Assembly of pH-sensitive liposome-coated M@BF (LM@BF) and HLM@BF

LM@BF was prepared by mixing Lipo (75 mg) with M@BF (75 mg) in 10 mL of ultrapure water, followed by sonication in an ice bath (200 W, 10 min) to facilitate lipid-bilayer coating onto the silica surface. The mixture was then stirred at room temperature for 30 min. HLM@BF was assembled using the same protocol, with HA-Lipo replacing Lipo. All formulations were filtered through a 0.22 μm sterile filter before in vitro and in vivo applications.

#### Physicochemical characterization and drug loading determination

TEM imaging was performed at an accelerating voltage of 200 kV using samples deposited onto carbon-coated copper grids. Energy-dispersive X-ray spectroscopy (EDS) coupled with scanning transmission electron microscopy (STEM) was used for elemental mapping, and surface elemental composition was quantified by X-ray photoelectron spectroscopy (XPS; ESCALAB 250Xi, Thermo Fisher Scientific). Hydrodynamic diameter, polydispersity index (PDI), and zeta potential were measured by DLS at 25 °C in triplicate. FTIR spectra (4000 –400 cm⁻¹) were recorded using the KBr pellet method. The drug loading (DL) and encapsulation efficiency (EE) of 5-FU and BMF-219 were determined by HPLC on a C18 column (250 mm × 4.6 mm, 5 μm) using methanol/water (80:20, v/v) as the mobile phase at a flow rate of 1 mL/min (25 °C) with a 20 µL injection volume. Detection wavelengths were 265 nm for 5-FU and 344 nm for BMF-219. Drug concentrations were calculated from external calibration curves generated using standard solutions of 5-FU and BMF-219 under the same HPLC conditions. DL was calculated as the mass of loaded drug divided by the total mass of the drug-loaded formulation, and EE was calculated as the mass of loaded drug divided by the initial mass of drug added. Cy5.5 loading was quantified by ultraviolet-visible (UV-Vis) spectroscopy.

#### Serum stability assay

To evaluate physiological stability, HLM@BF nanoparticles were dispersed in PBS containing 10% fetal bovine serum (FBS) and incubated at 37 °C under continuous shaking. The hydrodynamic diameter was monitored via DLS at predefined time points (1, 2, 3, 4, 5, 6, and 7 days).

#### In vitro tumor microenvironment (TME)-responsive degradation and drug release

The morphological degradation of HLM@BF under tumor microenvironment (TME)-mimicking conditions was observed by transmission electron microscopy (TEM). Nanoparticles were incubated in PBS (pH 7.4 or 5.0) with or without 10 mM glutathione (GSH) for 1 and 3 days. For release kinetics, HLM@BF nanoparticles were sealed in dialysis bags (molecular weight cutoff [MWCO], 3.5 kDa) and immersed in 50 mL of release medium at 37 °C: PBS (pH 7.4), PBS (pH 5.0), PBS (pH 7.4) containing 10 mM GSH, or PBS (pH 5.0) containing 10 mM GSH. At predetermined intervals, 1 mL of the external medium was withdrawn and replaced with an equal volume of fresh medium. Released 5-FU and BMF-219 were quantified by HPLC using calibration curves prepared under the same analytical conditions.

#### Cellular uptake, endo/lysosomal trafficking, and targeting mechanism

To evaluate the contribution of CD44 to nanoparticle uptake, a CD44 knockdown model was established in SNU-216 cells using siCD44 as described above. Untreated, negative-control siRNA-transfected, and siCD44-transfected cells were incubated with Cy5.5-labeled HLM@BF nanoparticles for 4 h. Cells were then harvested, and intracellular fluorescence intensity was quantified by flow cytometry using the same analysis settings across groups.

For morphological evaluation of cellular uptake, cells were incubated with Cy5.5-labeled HLM@BF for 1, 2, and 4 h, fixed, counterstained with DAPI, and imaged using a confocal laser scanning microscope (CLSM; Leica TCS SP8, Leica Microsystems, Germany). To track intracellular trafficking, cells treated with nanoparticles for 2, 6, and 12 h were co-stained with LysoTracker Green (Beyotime, Shanghai, China; Cat# C1047S) and DAPI. Images were acquired under identical settings within each experiment. The temporal colocalization between nanoparticles (red) and endo/lysosomes (green) was quantitatively analyzed by calculating Manders’ overlap coefficient (tM1) using ImageJ software (version 1.8.0, NIH, USA).

#### In vitro cytotoxicity and hemolysis assay

The in vitro biosafety of the blank nanocarriers, including M, LM, and HA-modified pH-sensitive liposome-coated M (HLM), was assessed using the CCK-8 assay over a concentration gradient (20–400 µg/mL) for 48 h. The cytotoxicity of the drug-loaded formulations, including LM@BF and HLM@BF, was evaluated under the same conditions.

To evaluate blood compatibility for potential intravenous administration, a hemolysis assay was performed. Fresh red blood cells (RBCs) were collected, washed, and incubated with varying concentrations of LM@BF and HLM@BF at 37 °C for 2 h. Pure water and saline served as the positive (100% hemolysis) and negative (0% hemolysis) controls, respectively. Following centrifugation, the absorbance of the supernatant was measured at 540 nm to calculate the hemolysis rate. The hemolysis rate was calculated according to the following equation:

$$\begin{aligned} \mathrm{Hemolysis}\:\left(\%\right)&=\left[\left(\mathrm{ODsample}-\mathrm{ODnegative}\right)\right. \\& \left./\left(\mathrm{ODpositive}-\mathrm{ODnegative}\right)\right]\\&\times{100}, \end{aligned}$$

where ODsample, ODnegative, and ODpositive represent the absorbance values of the nanoparticle-treated group, the saline negative control, and the pure water positive control, respectively. A hemolysis rate below 5% was considered to meet the commonly used biosafety threshold for intravenous administration.

#### Mechanism rescue and western blotting

To examine HLM@BF-induced apoptotic signaling under TP53 perturbation, SNU-216 cells were transfected with siRNA targeting TP53 (siTP53) or negative-control siRNA using Lipofectamine 3000 according to the manufacturer’s protocol. At 48 h post-transfection, knockdown efficiency was confirmed before HLM@BF treatment. For Western blotting, total protein was extracted using RIPA lysis buffer supplemented with protease and phosphatase inhibitors and quantified using a bicinchoninic acid (BCA) assay kit. Equal amounts of protein (30 µg) were separated by sodium dodecyl sulfate-polyacrylamide gel electrophoresis (SDS-PAGE), transferred onto polyvinylidene difluoride (PVDF) membranes, and blocked with 5% non-fat milk. The membranes were probed overnight at 4 °C with primary antibodies against Menin, p53, p-p53, Bcl-2, Bax, and ACTB. Following incubation with HRP-conjugated secondary antibodies, protein bands were visualized using an Enhanced Chemiluminescence Detection Kit. Band intensities were quantified using ImageJ and normalized to ACTB unless otherwise specified.

#### In vivo xenograft tumor model and treatment regimens

Male BALB/c nude mice (4–6 weeks old, approximately 15 g) were obtained from the Laboratory Animal Center of Guangxi Medical University and housed in a specific pathogen-free (SPF) facility. All animal procedures strictly adhered to the NIH Guide for the Care and Use of Laboratory Animals and were approved by the Institutional Animal Care and Use Committee of Guangxi Medical University. SNU-216 cells were selected for xenograft construction because this cell line showed lower drug IC50 values and a more pronounced BF-induced apoptotic response than MKN-28 in the preceding in vitro assays. The GC xenograft model was established via subcutaneous injection of SNU-216 cells (1 × 10⁶ cells/mouse) suspended in PBS into the right dorsal flank. Tumor dimensions were measured using digital calipers, and tumor volume (V) was calculated as $\mathrm{V}=0.5\times\mathrm{L}\times\mathrm{W}^{2}$, where L and W represent tumor length and width, respectively. Upon reaching a tumor volume of approximately 30 mm³, mice were randomized for subsequent interventions. No animal exceeded the maximum tumor burden permitted by the approved animal protocol.

To evaluate therapeutic efficacy, two treatment protocols were employed once tumor volumes reached approximately 30 mm³. The sample size (*n* = 4 or 5 per group) was based on preliminary feasibility experiments and prior studies while minimizing animal use. The paratumoral free-drug experiment was used as a preliminary proof-of-concept evaluation of the BF combination, whereas the intravenous study was used to evaluate the therapeutic performance of the nanoplatform formulations. For the preliminary free-drug evaluation, mice were randomly assigned to four groups (Control, BMF-219, 5-FU, and BF combination) using a random number table generator. Treatments were administered via paratumoral injections at a dose of 30 mg/kg for each drug, given every 3 days for a total of 4 doses.

For the targeted nanoplatform evaluation, mice were randomly assigned to four groups: saline, free BF (12 mg/kg; 5-FU: BMF-219 = 1:1), LM@BF (200 mg/kg nanoparticle equivalent), and HLM@BF (200 mg/kg nanoparticle equivalent). Based on the measured DL values in Table 1, the 200 mg/kg LM@BF dose corresponded to approximately 4.96 mg/kg 5-FU and 6.30 mg/kg BMF-219, whereas the 200 mg/kg HLM@BF dose corresponded to approximately 5.04 mg/kg 5-FU and 6.14 mg/kg BMF-219. Thus, the nanoparticle groups were broadly matched to the free BF dose in total payload exposure, while preserving the measured formulation-specific drug ratios. Treatments were administered intravenously by tail-vein injection every 3 days for a total of 5 doses (Days 0, 3, 6, 9, and 12). Tumor volumes and body weights were monitored concurrently. Mice were euthanized on Day 12 for the free-drug study or Day 18 for the nanoplatform study for subsequent histopathological evaluations.

**Table 1:** Drug loading (DL) and encapsulation efficiency (EE) of the nanocarriers

Formulation	5-FU DL % (EE %)	BMF-219 DL % (EE %)
M@BF	5.15 ± 0.04 (64.42 ± 0.23)	6.02 ± 0.04 (75.26 ± 0.40)
LM@BF	2.48 ± 0.05 (62.00 ± 0.44)	3.15 ± 0.05 (78.66 ± 0.30)
HLM@BF	2.52 ± 0.07 (63.14 ± 0.25)	3.07 ± 0.06 (76.93 ± 0.26)

Data are presented as mean ± SD, *n* = 3 independent preparation batches. DL = drug loading, calculated relative to total formulation mass at each assembly stage. EE = encapsulation efficiency, calculated relative to the initial input amount of each drug. The final HLM@BF injectable formulation contained 5-FU and BMF-219 at an approximate mass ratio of 1:1.22

#### In vivo biodistribution and fluorescence imaging

To investigate in vivo targeting capability and biodistribution, tumor-bearing mice were intravenously injected with Cy5.5-labeled nanocarriers at the same nanoparticle-equivalent dose used for the nanoplatform therapeutic evaluation (200 mg/kg). For the HLM@BF time-course study, major organs (heart, liver, spleen, lung, kidney, and brain) and tumors were excised at 1, 3, 6, 12, 24, and 48 h post-injection. For the targeted versus non-targeted comparison, Cy5.5-labeled LM@BF or HLM@BF was administered using the same nanoparticle-administration framework as the nanoplatform therapeutic evaluation, and tumors and major organs were collected for ex vivo fluorescence imaging at the corresponding endpoint. Whole-body imaging was used only as an auxiliary qualitative observation because fur, tissue depth, and overlap between adjacent organs can attenuate and confound fluorescence signals. Therefore, quantitative biodistribution analysis was based on ex vivo imaging of dissected tumors and major organs using an in vivo imaging system (IVIS) (PerkinElmer, USA; excitation, 675 nm; emission, 720 nm). Ex vivo fluorescence images were acquired using identical imaging settings within each experiment, and regions of interest were quantified as average radiant efficiency after background correction using Living Image software (version 4.4; PerkinElmer). This assay was designed to provide quantitative carrier-level biodistribution information rather than drug-level pharmacokinetic profiles of free or encapsulated 5-FU and BMF-219.

#### Systemic biosafety and histological evaluation

At the end of the treatment period, whole blood was collected for hematological analysis, including red blood cells (RBC), white blood cells (WBC), and platelets (PLT), using an automated veterinary hematology analyzer (BC-5000VET; Mindray, Shenzhen, China). Serum was isolated by centrifugation to evaluate biochemical markers of liver function (alanine aminotransferase, ALT; aspartate aminotransferase, AST) and kidney function (urea, UREA; creatinine, CREA) using an automated biochemical analyzer (BS-240VET; Mindray, Shenzhen, China). The excised major vital organs (heart, liver, spleen, lung, kidneys) and tumors were fixed in 4% paraformaldehyde, embedded in paraffin, sectioned, and stained with hematoxylin and eosin (H&E) for histopathological examination.

#### Immunohistochemistry (IHC) analysis

To validate the in vivo molecular mechanisms, paraffin-embedded tumor sections were subjected to immunohistochemical (IHC) staining. Sections were deparaffinized in xylene, rehydrated through a graded ethanol series, and subjected to heat-induced antigen retrieval. After blocking, sections were incubated overnight at 4 °C with primary antibodies targeting Menin, phosphorylated p53 (p-p53), total p53, Bcl-2, and Bax. After washing and secondary antibody incubation, immunoreactive signals were visualized using a 3,3’-diaminobenzidine (DAB) substrate kit. Nuclei were counterstained with hematoxylin, and representative images were captured by optical microscopy.

### Statistical analysis

All statistical analyses were conducted using SPSS 23.0 software (IBM, Armonk, NY, USA; RRID: SCR_002865). Quantitative data are expressed as the mean ± standard deviation (SD). Statistical comparisons between two independent groups were performed using Student’s t-test. For comparisons among multiple groups, one-way analysis of variance (ANOVA) followed by the indicated post-hoc test was used. Graphical representations were generated using GraphPad Prism 9.5. Bioinformatics analyses were performed in R (v4.3.3) using DESeq2 (v1.42.0) and clusterProfiler (v4.8.1). A P-value < 0.05 was considered to indicate statistically significant differences.

## Results

### Synergistic antitumor efficacy of BMF-219 and 5-FU in gastric cancer

**Fig. 1:**
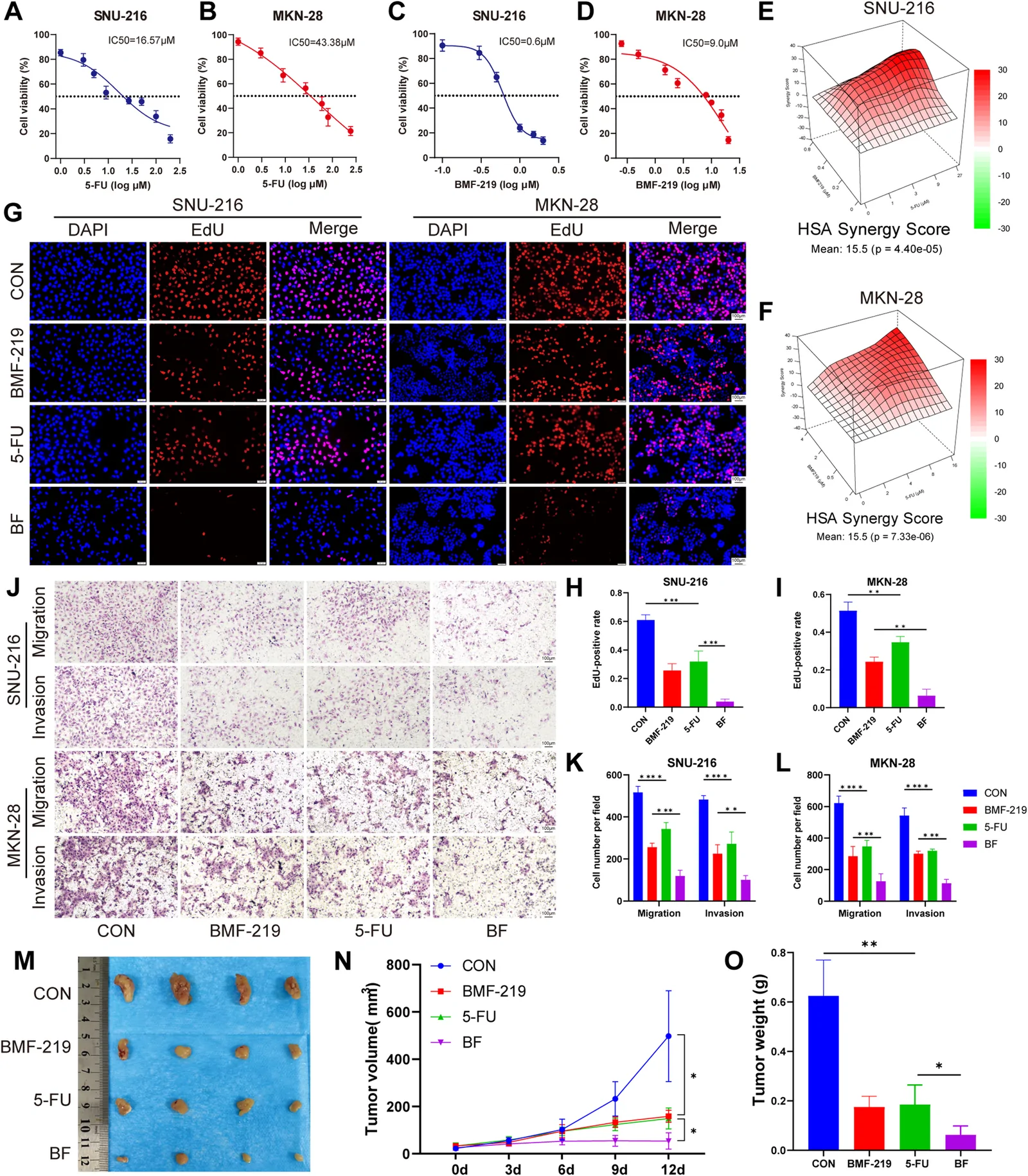
Synergistic antitumor effects of BMF-219 and 5-fluorouracil (5-FU) on gastric cancer models. **A**-**B**. In vitro cytotoxicity and half-maximal inhibitory concentration (IC50) determination of 5-FU in SNU-216 (**A**) and MKN-28 (**B**) cells after 48 h of treatment. **C**-**D**. In vitro cytotoxicity and IC50 determination of BMF-219 in SNU-216 (**C**) and MKN-28 (**D**) cells after 48 h of treatment. **E**-**F**. Three-dimensional synergy landscapes and synergy scores calculated using the Highest Single Agent (HSA) reference model in SynergyFinder for SNU-216 (**E**) and MKN-28 (**F**) cells treated with the BMF-219/5-FU combination (BF). A synergy score > 10 indicates synergistic interaction. **G**. Representative fluorescence images of 5-ethynyl-2’-deoxyuridine (EdU; red, proliferating cells) and 4’,6-diamidino-2-phenylindole (DAPI; blue, nuclei) staining after treatment with control, BMF-219, 5-FU, or BF in SNU-216 and MKN-28 cells. **H**-**I**. Quantification of the EdU-positive rate in SNU-216 (H) and MKN-28 (**I**) cells. **J**. Representative Transwell migration and invasion images after treatment with control, BMF-219, 5-FU, or BF in SNU-216 and MKN-28 cells. **K**-**L**. Quantification of migrated and invaded cells per field in SNU-216 (**K**) and MKN-28 (**L**) cells. **M**. Representative macroscopic images of excised SNU-216 subcutaneous xenograft tumors at Day 12 after treatment with control, BMF-219, 5-FU, or BF. **N**. Tumor volume growth curves during the therapeutic period. **O**. Average tumor weight at the experimental endpoint. Data are presented as mean ± standard deviation (SD) (n = 3 for in vitro assays; n = 4 for in vivo experiments). ^*^P < 0.05, ^**^P < 0.01, ^***^P < 0.001, ****P < 0.0001

To evaluate the therapeutic potential of co-administering 5-fluorouracil (5-FU) and the Menin inhibitor BMF-219, we first determined their respective IC50 in gastric cancer cell lines SNU-216 and MKN-28. The IC50 values of 5-FU were 16.57 µM for SNU-216 and 43.38 µM for MKN-28 (Fig. 1A, B). For BMF-219, the IC50 values were 0.6 µM in SNU-216 and 9.0 µM in MKN-28 (Fig. 1C, D), indicating potent intrinsic cytotoxicity.

We then treated cells with a dose-combination matrix of 5-FU and BMF-219 (denoted as BF). Synergy analysis utilizing the HSA reference model in the SynergyFinder platform revealed robust synergistic inhibition in both cell lines, yielding high synergy scores of 15.5 for both SNU-216 and MKN-28 (Fig. 1E, F).

We subsequently assessed the impact of the BF combination on cell proliferation and metastasis. The EdU incorporation assay demonstrated that the BF combination significantly suppressed DNA replication, resulting in a significantly lower percentage of EdU-positive proliferating cells compared to the control and monotherapy groups (Fig. 1G–I). Furthermore, Transwell assays revealed that the BF combination markedly impaired the motility of GC cells, as shown by a reduction in both migrated and invaded cells (Fig. 1J–L).

We next examined the molecular basis of BMF-219 activity by assessing Menin protein stability. Because BMF-219 is a covalent Menin inhibitor, we evaluated whether BMF-219 treatment was associated with altered Menin abundance in addition to functional inhibition. Immunoprecipitation and ubiquitination assays showed that BMF-219 reduced total Menin protein in input lysates, whereas co-treatment with the proteasome inhibitor MG-132 attenuated this reduction (Fig. S1A). Immunoprecipitation further revealed an accumulation of high-molecular-weight polyubiquitinated Menin species in cells co-treated with BMF-219 and MG-132. These data support an association between BMF-219 treatment, reduced Menin abundance, and increased ubiquitinated Menin species under the tested conditions.

To translate these in vitro findings, we evaluated the therapeutic efficacy of the free drug combination in a SNU-216 subcutaneous xenograft model. Over a 12-day therapeutic window, the free BF combination group exhibited the most potent inhibition of tumor proliferation (Fig. 1N). At the experimental endpoint, the excised tumors in the BF cohort were visibly the smallest (Fig. 1M), and the average tumor weight was significantly lower than those in the single-agent groups (*P* < 0.05 versus BMF-219 monotherapy and 5-FU monotherapy; *P* < 0.01 versus Control) (Fig. 1O).

Collectively, these in vitro and in vivo evaluations support a synergistic antitumor effect of co-administered BMF-219 and 5-FU.

### The BF combination synergistically induces apoptosis through Menin- and p53-associated apoptotic signaling

**Fig. 2:**
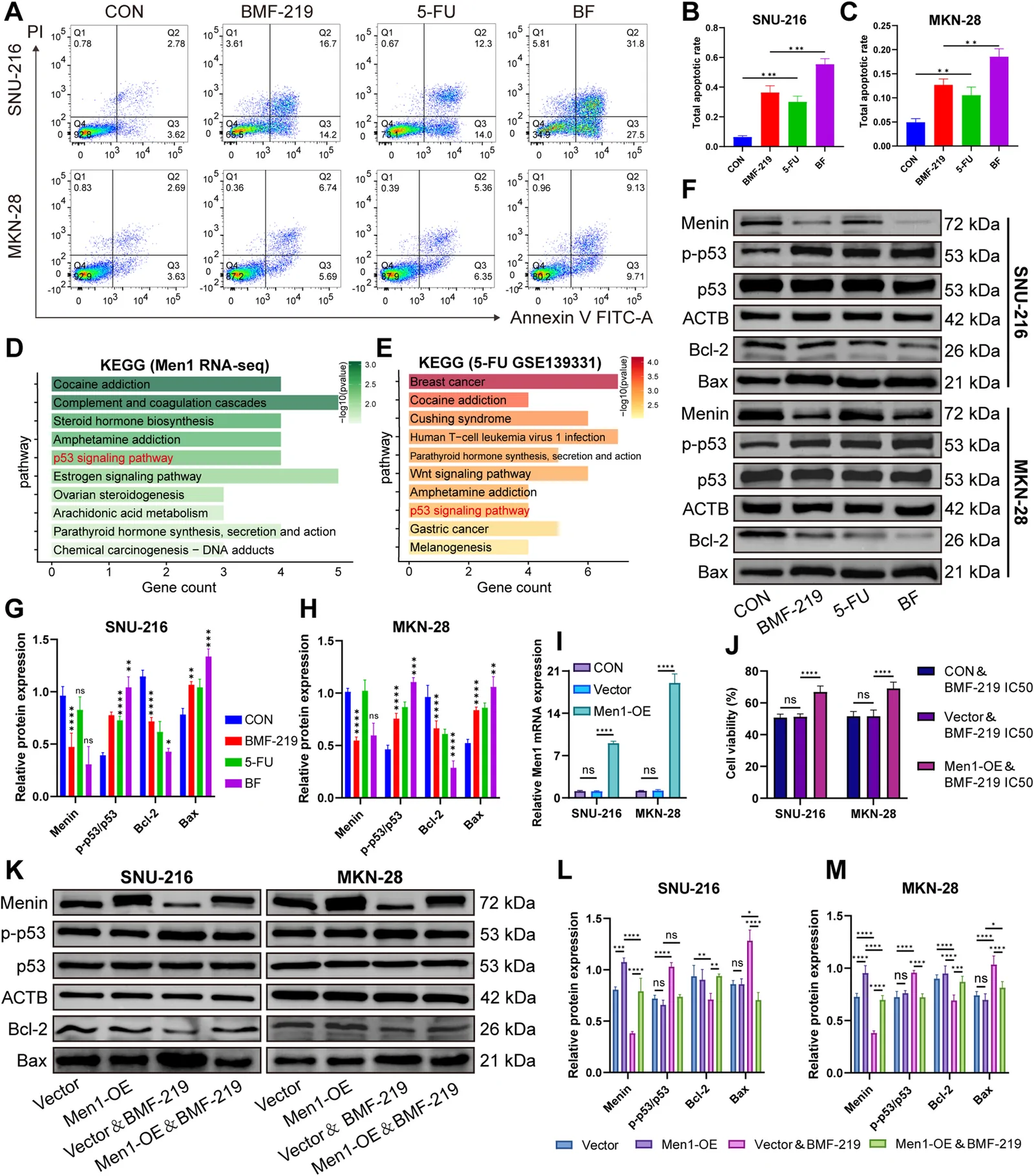
The BF combination induces apoptosis through Menin- and p53-associated apoptotic signaling. **A**. Representative flow cytometry plots of Annexin V-fluorescein isothiocyanate/propidium iodide (Annexin V-FITC/PI) dual staining after 48 h of treatment with control, BMF-219, 5-FU, or BF in SNU-216 and MKN-28 cells. **B**-**C**. Quantification of the total apoptotic cell rate, including early and late apoptotic populations, in SNU-216 (**B**) and MKN-28 (**C**) cells. **D**. Kyoto Encyclopedia of Genes and Genomes (KEGG) pathway enrichment analysis based on our previously generated in-house RNA sequencing (RNA-seq) dataset from Men1-overexpressing (Men1-OE) cells. **E**. KEGG pathway enrichment analysis of differentially expressed genes in 5-FU-treated gastric cancer cells from the GSE139331 dataset. **F**. Representative Western blot images of Menin, phosphorylated p53 (p-p53), total p53, Bcl-2, and Bax in SNU-216 and MKN-28 cells treated with control, BMF-219, 5-FU, or BF. ACTB was used as the loading control. **G**-**H**. Quantitative analysis of protein expression in SNU-216 (**G**) and MKN-28 (**H**) cells. **I**. Verification of Men1 overexpression efficiency by quantitative real-time polymerase chain reaction (qRT-PCR). **J**. Cell viability of Vector control and Men1-OE cells after treatment with BMF-219 at the corresponding IC50 concentration. **K**. Western blot analysis of Menin- and apoptosis-related markers in Vector and Men1-OE cells after BMF-219 treatment. **L**-**M**. Quantitative analysis of protein expression in SNU-216 (**L**) and MKN-28 (**M**) cells. Data are presented as mean ± SD (n = 3). ^*^P < 0.05, ^**^P < 0.01, ^***^P < 0.001, ****P < 0.0001; ns, not significant

To determine the cellular outcome underlying the observed synergistic tumor suppression, we performed Annexin V-FITC/PI dual-staining flow cytometry. The results demonstrated that the BF combination triggered substantial cellular apoptosis compared with the control and monotherapy groups (Fig. 2A). Quantitative analysis revealed that the total apoptosis rates in the synergistic BF group reached approximately 59.3% in SNU-216 cells and 18.8% in MKN-28 cells, both of which were significantly higher than those induced by single-agent treatments (Fig. 2B, C).

To evaluate the molecular basis associated with this synergistic apoptosis, we first performed bioinformatic screening. Data retrieved from CTD identified TP53 as the gene most strongly associated with fluorouracil among the top interacting genes (Fig. S1B). GEPIA2 analysis further revealed a significant positive correlation between TP53 and Men1 expression in GC (*R* = 0.32, *P* = 2.2e-16; Fig. S1C). In addition, TCGA-STAD analysis through UCSC Xena showed that Men1 expression was significantly higher in tumor tissues than in normal tissues (*P* = 1.6e-08; Fig. S1D). Guided by these findings, we analyzed our previously generated in-house RNA-seq dataset from Men1-overexpressing (Men1-OE) cells and performed pathway enrichment analysis. KEGG pathway enrichment of DEGs derived from this dataset highlighted the p53 signaling pathway (Fig. 2D). Concurrently, parallel analysis of the GSE139331 dataset (5-FU-treated GC cells) also identified the p53 signaling pathway as a primary enriched cascade (Fig. 2E). This transcriptomic convergence supports the p53 regulatory network as a major pathway engaged by Menin inhibition and 5-FU treatment.

We subsequently validated these transcriptomic findings at the translational level. Western blot analysis revealed that treatment with the BF combination resulted in a marked downregulation of Menin, coupled with a marked upregulation of phosphorylated p53 (p-p53) and its downstream pro-apoptotic effector, Bax (Fig. 2F–H). Concurrently, the expression of the anti-apoptotic protein Bcl-2 was intensely suppressed.

To test whether Menin inhibition contributed functionally to the observed apoptosis and molecular changes, we performed gain-of-function rescue experiments using stable Men1-OE cell lines (Fig. 2I). Under BMF-219 treatment (at IC50 concentrations), the cell viability of the Men1-OE group remained significantly higher than that of the Vector control group (Fig. 2J), indicating that restoring intracellular Menin levels partly counteracted the inhibitor-associated cytotoxicity. At the molecular level, the ability of BMF-219 to upregulate p-p53/Bax and downregulate Bcl-2 was weakened in the Men1-OE group compared to the Vector group (Fig. 2K–M).

Collectively, these rescue data support the interpretation that BMF-219 promotes pro-apoptotic sensitization at least partly through Menin inhibition and p53-associated apoptotic signaling.

### Synthesis, physicochemical characterization, and drug loading profile of the HLM@BF nanoplatform

**Fig. 3:**
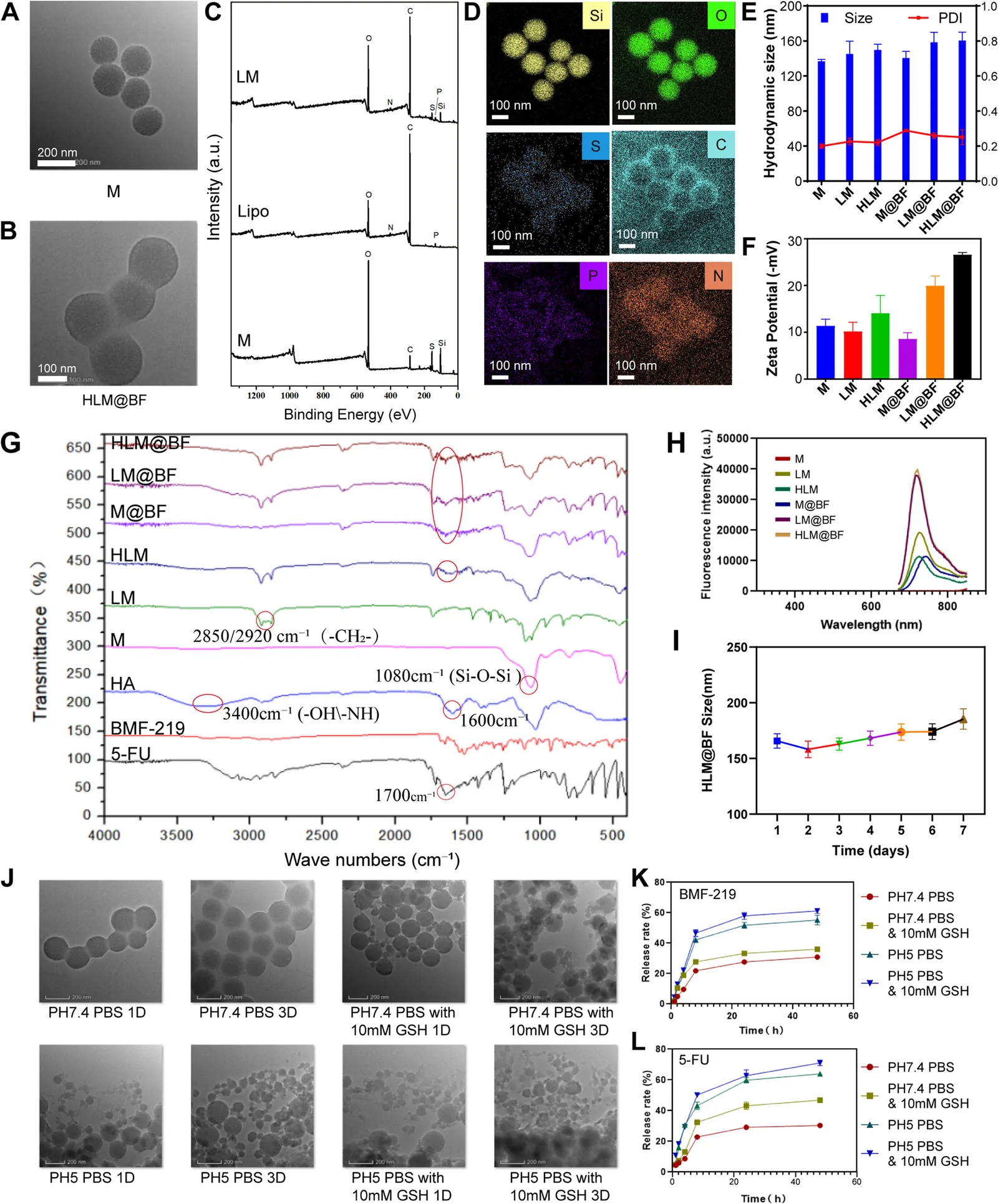
Synthesis, physicochemical characterization, and tumor microenvironment (TME)-responsive properties of HLM@BF. **A**-**B**. Representative transmission electron microscopy (TEM) images of disulfide-bridged mesoporous silica nanoparticles (**M**) (**A**) and the final hyaluronic acid-decorated liposome/mesoporous silica nanoplatform loaded with BMF-219 and 5-FU (HLM@BF) (**B**), showing the morphology of the silica core and core-shell architecture. **C**. X-ray photoelectron spectroscopy (XPS) survey spectra of M, pH-sensitive liposomes (Lipo), liposome-coated M (LM), showing changes in surface elemental composition during stepwise assembly. **D**. Energy-dispersive X-ray spectroscopy (EDS) elemental mapping of HLM@BF, showing the spatial distribution of Si, O, S, C, N, and P signals. **E**. Hydrodynamic diameter and polydispersity index (PDI) of different formulations measured by dynamic light scattering (DLS). **F**. Zeta potential changes during nanoparticle assembly. **G**. Fourier transform infrared spectroscopy (FTIR) spectra of 5-FU, BMF-219, hyaluronic acid (HA), M, HLM, M@BF, LM@BF, and HLM@BF, supporting successful drug loading, lipid coating, and HA decoration. **H**. Fluorescence emission spectra of unloaded and Cy5.5-labeled nanoparticle formulations. **I**. Colloidal stability of HLM@BF in serum-containing medium over 7 days, evaluated by hydrodynamic size changes. **J**. TEM images showing morphological degradation of HLM@BF under simulated TME conditions, including acidic pH and glutathione (GSH). **K**-**L**. Cumulative release profiles of BMF-219 (**K**) and 5-FU (**L**) from HLM@BF under different pH and GSH conditions. Data are presented as mean ± SD (*n* = 3)

To reduce systemic toxicity and improve the targeted accumulation of the synergistic BMF-219/5-FU combination, we engineered a hyaluronic acid (HA)-decorated, pH-sensitive liposome-coated mesoporous silica nanoplatform (HLM@BF). The morphological evolution of the nanocarriers was tracked by TEM. The bare mesoporous silica cores (M) exhibited a highly uniform, spherical morphology with a distinct porous structure (Fig. 3A). Following dual-drug loading, Cy5.5 incorporation, encapsulation within the pH-sensitive DOPE/CHEMS lipid shell, and final modification with 50 kDa HA, the HLM@BF nanoparticles maintained high dispersity. A distinct organic thin film surrounding the dense silica cores was visible, supporting successful hierarchical assembly (Fig. 3B).

XPS survey spectra showed that LM displayed dominant C/O signals and detectable N/P signals associated with the lipid layer, while Si and S signals from the silica/disulfide framework remained detectable within the XPS sampling depth (Fig. 3C). This pattern supports lipid-associated surface modification of the silica-based core, but should not be interpreted as molecularly complete surface coverage. EDS elemental mapping further showed Si/O/S signals associated with the silica/disulfide framework together with C/N/P signals distributed around the nanoparticle architecture (Fig. 3D). Together with the TEM-observed organic layer, these results support formation of a liposome-associated shell rather than simple particle aggregation.

FTIR spectroscopy provided additional evidence for stepwise assembly (Fig. 3G). The bare M exhibited Si-O-Si stretching at 1080 cm⁻¹; liposome coating introduced -CH2- stretching at 2850/2920 cm⁻¹; and HA decoration was supported by the broad -OH/-NH band at 3400 cm⁻¹ and carboxylate stretching at 1600 cm⁻¹. DLS measurements showed an incremental size increase from approximately 135 nm for M to 160 nm for HLM@BF, with PDI values consistently below 0.3 (Fig. 3E). Zeta potential measurements showed a stepwise surface charge shift, reaching − 26.9 mV for HLM@BF, consistent with the introduction of carboxyl-rich HA on the outer surface (Fig. 3F).

To determine carrier capacity, the encapsulation efficiency (EE) and drug loading (DL) at different assembly stages were quantified (Table 1). EE was calculated relative to the initial input amount of each drug, whereas DL was calculated relative to the total formulation mass at each assembly stage. This distinction explains why DL decreased after lipid/HA coating while EE values remained within a comparable range across the assembly stages. The final injectable HLM@BF formulation contained 5-FU and BMF-219 at a mass ratio of approximately 1:1.22, with an EE of 63.14% (DL: 2.52%) for 5-FU and 76.93% (DL: 3.07%) for BMF-219.

Prior to biological evaluation, optical integrity and colloidal stability were assessed. Fluorescence spectroscopy (Fig. 3H) confirmed that Cy5.5 was incorporated into the nanocarriers, a prerequisite for optical tracking. Furthermore, when incubated in 10% FBS-supplemented PBS, HLM@BF exhibited negligible size fluctuations over a 7-day monitoring period (Fig. 3I), supporting favorable colloidal stability in a serum-containing medium.

### Tumor microenvironment (TME)-responsive degradation and drug release kinetics

A core design rationale of HLM@BF is its ability to spatiotemporally dismantle in response to the specific biochemical cues of the TME: the acidic endolysosomal pH and the highly reducing (elevated GSH) cytosolic environment. This responsive structural collapse was visualized via TEM (Fig. 3J). While the nanoparticles remained largely intact in neutral PBS (pH 7.4) for up to 3 days, exposure to a simulated acidic condition (pH 5.0) induced noticeable morphological loosening, reflecting the destabilization of the DOPE/CHEMS lipid shell. The addition of 10 mM GSH provoked severe degradation; under dual-stimuli conditions (pH 5.0 + 10 mM GSH), the core-shell architecture was extensively fragmented by day 3.

This morphologic disintegration was consistent with the drug release kinetics. In vitro release profiles of both BMF-219 (Fig. 3K) and 5-FU (Fig. 3L) displayed limited premature leakage (< 30% over 48 h) under physiological conditions (pH 7.4). Conversely, the highest release rates were observed under the combined TME-mimicking conditions (pH 5.0 + 10 mM GSH), wherein approximately 60% of BMF-219 and 70% of 5-FU were liberated within 48 h. This dual-responsive profile supports preferential release under TME-mimicking conditions while limiting premature leakage under neutral conditions.

### In vitro uptake, intracellular trafficking, biosafety, and mechanistic validation of HLM@BF

**Fig. 4:**
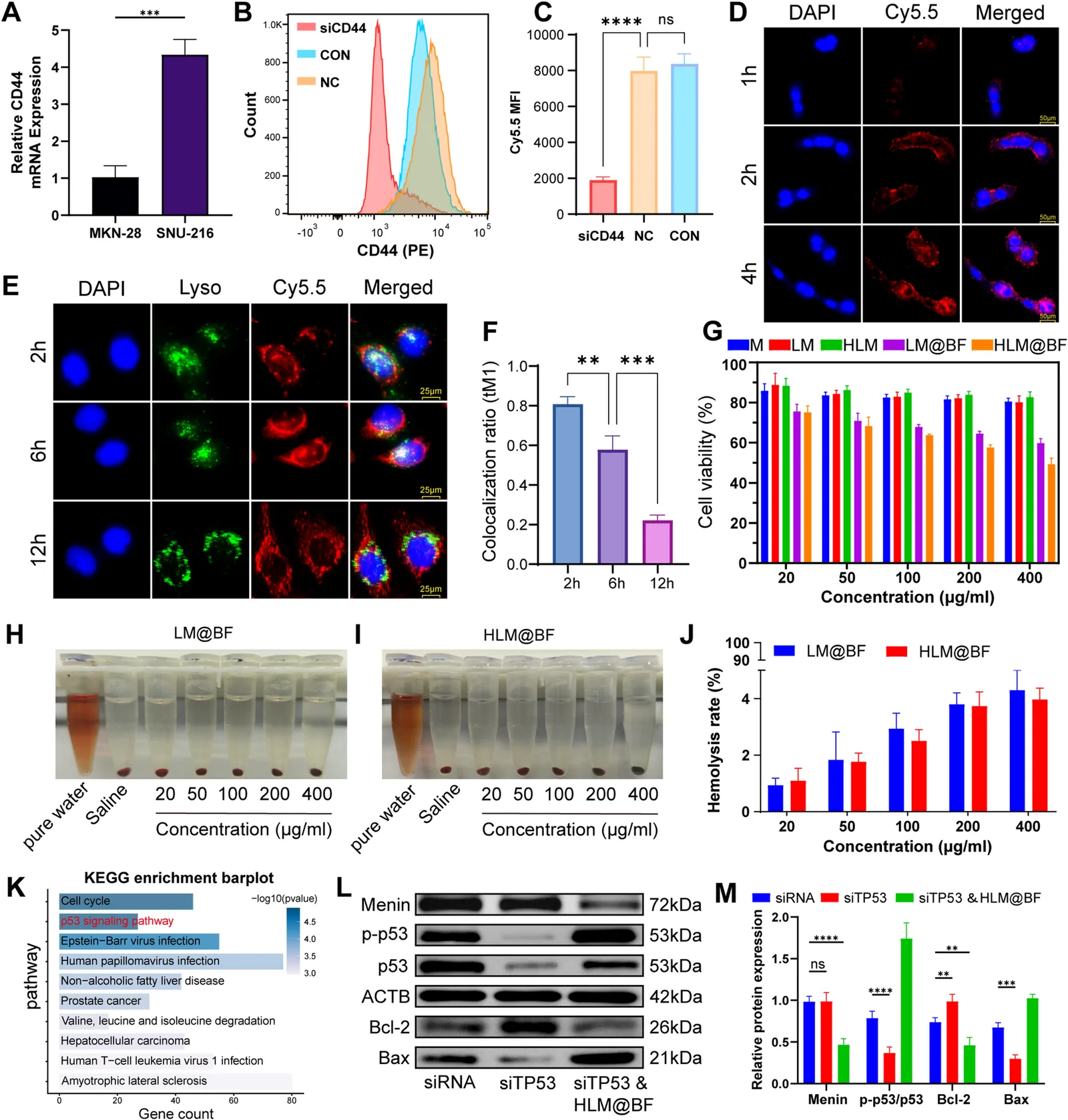
In vitro uptake, intracellular trafficking, biosafety, and mechanistic validation of HLM@BF. **A**. qRT-PCR analysis of basal CD44 mRNA expression in MKN-28 and SNU-216 gastric cancer cells. **B**. Flow cytometric confirmation of surface CD44 reduction after siCD44 transfection in SNU-216 cells. **C**. Mean fluorescence intensity (MFI) quantification of Cy5.5-labeled HLM@BF uptake in untreated, negative-control siRNA-transfected, and siCD44-transfected SNU-216 cells, evaluating the contribution of CD44 to nanoparticle internalization. **D**. Confocal laser scanning microscopy (CLSM) images showing time-dependent cellular uptake of Cy5.5-labeled HLM@BF in SNU-216 cells over 4 h. Nuclei were counterstained with DAPI. Scale bars: 50 μm. **E**. CLSM images showing intracellular trafficking of Cy5.5-labeled HLM@BF and LysoTracker-labeled endo/lysosomes at different time points. Nanoparticles are shown in red, endo/lysosomes in green, and nuclei in blue. Scale bars: 25 μm. **F**. Quantification of nanoparticle-lysosome colocalization using Manders’ coefficient. **G**. Cell viability of SNU-216 cells treated with blank vehicles, including M, LM, and HA-decorated LM (HLM), or drug-loaded formulations, including LM@BF and HLM@BF, for 48 h. **H**-**I**. Representative photographs of the hemolysis assay for red blood cells treated with LM@BF (**H**) or HLM@BF (**I**) at increasing concentrations. **J**. Quantitative hemolysis rates calculated from supernatant absorbance, with 5% used as the biosafety threshold for intravenous administration. **K**. KEGG pathway enrichment analysis based on RNA-seq of HLM@BF-treated SNU-216 cells versus normally cultured SNU-216 controls, highlighting enrichment of the p53 signaling pathway. **L**. Western blot analysis of Menin, p-p53, total p53, Bcl-2, and Bax in siNC, siTP53, and siTP53 plus HLM@BF-treated SNU-216 cells. **M**. Quantitative analysis of protein expression from panel **L**, showing HLM@BF-associated regulation of Menin- and p53-associated apoptotic markers under siTP53-transfected conditions. Data are presented as mean ± SD (*n* = 3). ^*^P < 0.05, ^**^P < 0.01, ^***^P < 0.001, ^****^P < 0.0001; ns, not significant

Following the transcriptomic and molecular analyses in both GC cell lines (Figs. 1 and 2), selecting an appropriate cellular model for evaluating the HA-decorated nanoplatform was necessary. Because the HLM@BF vehicle uses HA as a targeting ligand, CD44 abundance may influence uptake efficiency. We first evaluated basal CD44 mRNA expression in SNU-216 and MKN-28 cells. qRT-PCR analysis (Fig. 4A) showed that SNU-216 cells expressed higher CD44 mRNA than MKN-28 cells. Consequently, SNU-216 was selected as the primary in vitro model for subsequent nanoplatform evaluations. Flow cytometry further confirmed surface CD44 expression in SNU-216 cells and was used to verify CD44 reduction after siCD44 transfection (Fig. 4B).

To test the functional contribution of HA-CD44 interaction to nanoparticle uptake, we employed a loss-of-function approach using siRNA-mediated knockdown of CD44. As demonstrated by the flow cytometric histograms, transfection with siCD44 resulted in a clear leftward shift in fluorescence intensity, confirming reduced CD44 abundance on the cell surface (Fig. 4B). Subsequently, the cellular uptake efficiency was quantitatively compared. Flow cytometric analysis revealed that the mean fluorescence intensity (MFI) of Cy5.5-labeled HLM@BF in the CD44-depleted cells was markedly lower than that in the negative control (NC) and untreated SNU-216 cells (Fig. 4C). These functional data support a contribution of HA-CD44 interaction to HLM@BF internalization. CLSM imaging was then used to monitor time-dependent uptake in SNU-216 cells, showing a progressive increase in the intracellular Cy5.5 signal from 1 to 4 h (Fig. 4D).

After cellular uptake, endo/lysosomal escape is needed for the drugs to reach cytosolic and nuclear targets. We dynamically tracked this intracellular trafficking utilizing LysoTracker Green (Fig. 4E). At an early time point (2 h), the red fluorescence of the nanoparticles heavily overlapped with the green fluorescence of the lysosomes, generating a prominent yellow signal. However, as the incubation extended to 12 h, the red and green signals distinctly separated, consistent with endo/lysosomal escape associated with destabilization of the pH-sensitive DOPE/CHEMS lipid shell. This intracellular redistribution was quantitatively supported by a sharp, statistically significant decline in the Manders’ colocalization coefficient (tM1) from 0.81 at 2 h to 0.22 at 12 h (Fig. 4F).

We subsequently evaluated the biological effects of these formulations. The CCK-8 assay (Fig. 4G) showed that the blank vehicles (M, LM, HLM) maintained high cell viability (> 80%) even at elevated concentrations, supporting favorable material biocompatibility. Drug-loaded HLM@BF produced dose-dependent cytotoxicity against SNU-216 cells and outperformed non-targeted LM@BF. Furthermore, a hemolysis assay was conducted to assess compatibility with intravenous administration (Fig. 4H-J). Across all tested concentrations, both LM@BF and HLM@BF maintained hemolysis rates below the commonly used safety threshold of 5%, supporting favorable hemocompatibility.

Finally, to examine whether the cytotoxicity of HLM@BF was associated with Menin- and p53-related apoptotic signaling, we integrated transcriptomic mapping and molecular rescue experiments. RNA-seq analysis of HLM@BF-treated SNU-216 cells compared with normally cultured SNU-216 controls (*n* = 3 biological replicates per group) showed enrichment of the p53 signaling pathway among DEGs (Fig. 4K). To further examine p53-associated apoptotic signaling under TP53 perturbation, SNU-216 cells were transfected with siTP53 and then treated with HLM@BF. Compared with siTP53 alone, HLM@BF treatment decreased Menin and Bcl-2 expression and increased the p-p53/total p53 ratio and Bax expression (Fig. 4L, M). These results indicate that HLM@BF engages Menin- and p53-associated apoptotic signaling in a p53-perturbed cellular context, supporting the involvement of this pathway in the HLM@BF response.

### In vivo biodistribution and target accumulation profile

**Fig. 5:**
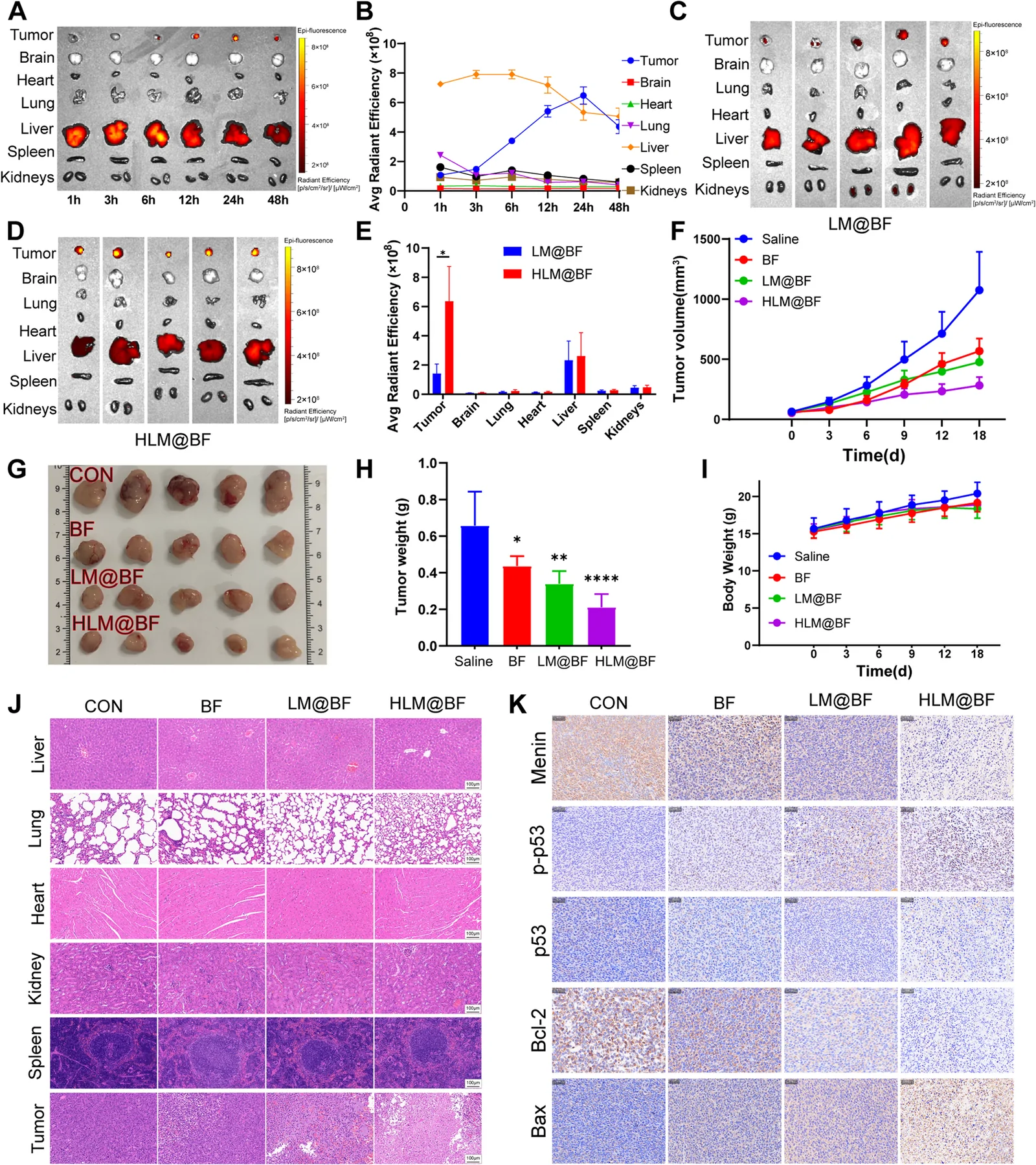
In vivo biodistribution, antitumor efficacy, and biosafety evaluation of HLM@BF. **A**. Time-dependent ex vivo fluorescence imaging of tumors and major organs, including brain, heart, lung, liver, spleen, and kidney, from SNU-216 xenograft-bearing mice after intravenous injection of Cy5.5-labeled HLM@BF. **B**. Quantitative analysis of average radiant efficiency in tumors and major organs over time. **C**-**D**. Representative ex vivo fluorescence images of tumors and major organs collected at the Day 18 endpoint after repeated intravenous administration of Cy5.5-labeled LM@BF (**C**) or HLM@BF (**D**), comparing non-HA-decorated and HA-decorated nanoplatforms. **E**. Quantification of average radiant efficiency in tumors and major organs for LM@BF and HLM@BF at the Day 18 endpoint. **F**. Tumor volume growth curves during the treatment period in mice treated with saline, free BF, LM@BF, or HLM@BF. **G**. Representative macroscopic images of excised tumors at Day 18. **H**. Average tumor weights at the experimental endpoint. **I**. Body weight changes during the therapeutic window. **J**. Hematoxylin and eosin (H&E) staining of major organs and tumor tissues, showing systemic tissue morphology and tumor necrosis after treatment. Scale bars: 100 μm. **K**. Immunohistochemistry (IHC) staining of Menin, p-p53, total p53, Bcl-2, and Bax in tumor tissues from different treatment groups. Scale bars: 50 μm. Data are presented as mean ± SD (n = 3 per time point for the time-course biodistribution analysis in panels A–B; n = 5 per group for the endpoint biodistribution comparison in panels C–E and the therapeutic experiments in panels F–K). ^*^P < 0.05, ^**^P < 0.01, ^***^P < 0.001, ^****^P < 0.0001

Efficient accumulation within the solid tumor microenvironment is a fundamental prerequisite for nanomedicine efficacy. We systematically evaluated the in vivo biodistribution of the Cy5.5-labeled nanoplatforms in SNU-216 xenograft-bearing mice. Ex vivo imaging of major organs and tumors harvested at sequential time points post-injection demonstrated a distinct spatiotemporal distribution of HLM@BF (Fig. 5A). Initially, prominent fluorescence was observed in the liver, reflecting standard reticuloendothelial system (RES) capture. However, consistent with EPR-associated tumor accumulation and HA-enhanced delivery, the tumor fluorescence progressively intensified, peaking between 12 h and 24 h, and retaining a strong signal even at 48 h (Fig. 5B).

**Fig. 6:**
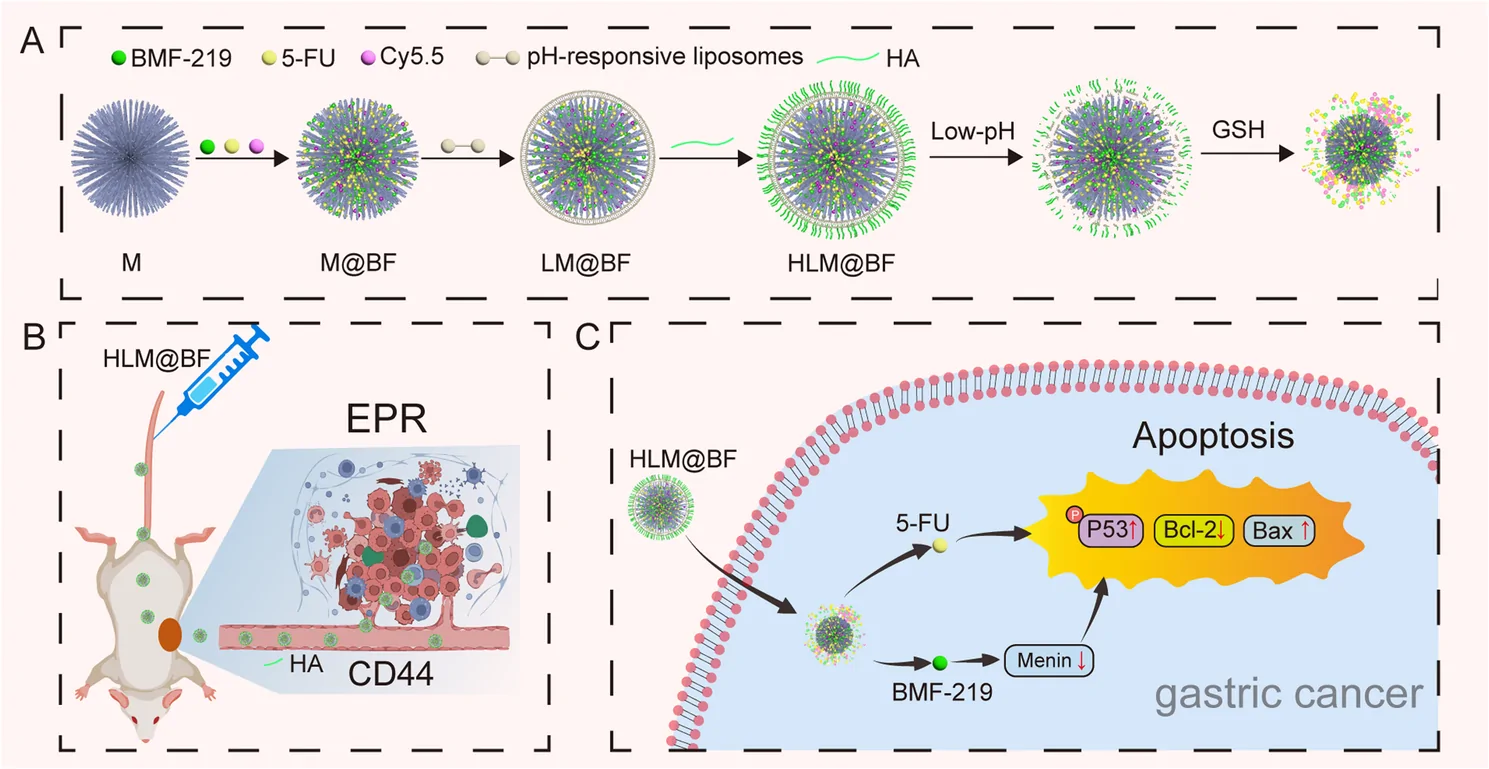
Schematic illustration of the fabrication and proposed mechanisms of the multistage stimuli-responsive HLM@BF nanoplatform for targeted synergistic therapy of gastric cancer. **A**. Layer-by-layer assembly of HLM@BF, including drug loading into the disulfide-bridged mesoporous silica core, coating with pH-sensitive liposomes, and surface decoration with HA. The nanoplatform is designed to reduce premature leakage and promote cascade-responsive drug release under acidic and GSH-rich intracellular conditions. **B**. Proposed in vivo delivery process, in which tumor accumulation is promoted by the enhanced permeability and retention (EPR) effect and HA-CD44-associated uptake. **C**. Proposed intracellular mechanism of synergistic antitumor efficacy. After cellular uptake and intracellular release, BMF-219 modulates Menin- and p53-associated apoptotic signaling, while 5-FU induces DNA damage; together, these effects promote mitochondrial apoptotic signaling characterized by altered p-p53, Bcl-2, and Bax expression

To evaluate the contribution of HA modification to tumor accumulation under the therapeutic administration setting, we compared the ex vivo biodistribution of non-HA-decorated LM@BF and HA-decorated HLM@BF after nanoparticle administration (Fig. 5C, D). Quantitative analysis of average radiant efficiency showed higher tumor accumulation for HLM@BF than for LM@BF (Fig. 5E). Together with the in vitro CD44-knockdown data, these findings support HA-enhanced tumor delivery and provide a mechanistic basis for the improved therapeutic performance of HLM@BF (Fig. 6).

### Robust synergistic antitumor efficacy and biosafety in vivo

SNU-216 xenografts were used for the in vivo evaluation because SNU-216 cells showed higher drug sensitivity and a markedly stronger BF-induced apoptotic response than MKN-28 cells in vitro. Guided by the favorable tumor-homing capability of HLM@BF, we subsequently assessed its in vivo therapeutic efficacy. Over the 18-day treatment window, tumors in the saline group grew rapidly. While the free BF combination and non-targeted LM@BF groups exhibited moderate tumor suppression, the targeted HLM@BF formulation achieved the most pronounced inhibition of tumor volume trajectory (Fig. 5F). At the experimental endpoint, the excised tumors visually corroborated this outcome (Fig. 5G), with the HLM@BF group presenting the smallest tumor mass and the lowest average tumor weight, indicating a statistically significant improvement in antitumor efficacy (Fig. 5H).

Achieving high efficacy must not compromise biological safety. Throughout the regimen, the body weights of mice in the HLM@BF group remained stable, closely mirroring the saline cohort (Fig. 5I), indicating an absence of severe cachexia or acute toxicity. To evaluate systemic biosafety, comprehensive hematological and biochemical assays were conducted (Fig. S1J–P). Hematological indices (RBC, PLT) and renal function markers (UREA, CREA) remained within normal physiological ranges across all groups. However, hepatic markers showed safety-related differences: mice administered with non-targeted LM@BF exhibited notable elevations in serum ALT, AST, and WBC levels, suggesting mild off-target hepatic stress that may be related to formulation-dependent hepatic interaction and reticuloendothelial system exposure. In contrast, the HA-decorated HLM@BF formulation largely avoided these changes, maintaining hepatic markers at healthy baseline levels. Histological H&E staining of major organs (heart, liver, spleen, lung, kidney) further confirmed the absence of structural abnormalities, inflammatory infiltration, or necrotic lesions in the HLM@BF group (Fig. 5J). Together, these data indicate that HA-enhanced delivery improved antitumor efficacy while reducing detectable systemic off-target toxicity in this model.

### In vivo epigenetic remodeling of the p53 pathway

To examine whether the in vivo antitumor effect was accompanied by modulation of the Menin/p53 axis, pathological and molecular examinations of the excised tumors were performed. H&E staining of the tumor tissues revealed that, compared to the dense viable cells in the control group, the HLM@BF-treated tumors exhibited the most extensive regions of severe necrosis and structural destruction (Fig. 5J).

Furthermore, immunohistochemical (IHC) profiling was consistent with the in vitro mechanistic findings (Fig. 5K). The HLM@BF nanoplatform reduced Menin expression within the tumor parenchyma. Consistent with our hypothesis, this was accompanied by increased expression of phosphorylated p53 (p-p53) and its pro-apoptotic effector Bax, together with reduced expression of the anti-apoptotic protein Bcl-2. Total p53 levels remained stable across all groups. These in vivo molecular data support the conclusion that HLM@BF enhances antitumor efficacy in association with Menin- and p53-related mitochondrial apoptotic signaling.

## Discussion

Our findings suggest that aberrant Menin activity may raise the apoptotic threshold by restraining p53-associated signaling in gastric cancer (GC). By introducing BMF-219, a clinical-stage covalent inhibitor of Menin, we effectively disrupted this molecular blockade. Using complementary in vitro assays and an in vivo xenograft model, our study supports Menin as a contributor to GC chemoresistance. Together, these findings support the rationale for combining Menin inhibition with 5-fluorouracil (5-FU) therapy and provide a biological basis for the engineered nanodelivery strategy.

A central conceptual contribution of our study is the characterization of Menin- and p53-associated apoptotic signaling in GC. This finding is consistent with recent interest in Menin inhibitors as a therapeutic class [16]. Traditionally, Menin has been regarded as a tumor suppressor in endocrine tumor biology, and recent work further shows that dysregulated Men1 degradation can promote pancreatic neuroendocrine tumor progression [23]. However, Menin increasingly exhibits a paradoxical, context-dependent oncogenic role in various other malignancies, including hepatocellular carcinoma, colorectal cancer, and GC [10, 24]. For instance, recent evidence indicates that CAF-derived exosomal Menin actively promotes GC progression within the tumor microenvironment [11]. In addition, a recent study reported that hsa-miR-149-5p targets Men1 to inhibit gastric cancer progression through the HSPA6/JNK pathway, further supporting the biological relevance of Men1-centered regulation in gastric cancer [25]. While a previous report suggested a contradictory tumor-suppressive function of Menin in GC via IQGAP1 interaction [26], this apparent discrepancy may reflect differences in cellular context, experimental endpoints, and the specific biological processes examined. Previous conclusions predominantly relied on basic in vitro proliferation assays, without comprehensive evaluations of metastatic phenotypes and in vivo validation. In contrast, our rescue experiments utilizing Men1-overexpressing cell lines supported the functional importance of Menin inhibition in the synergistic efficacy of the BF combination. Menin inhibition appears to shift the DNA damage response and the Bcl-2/Bax balance toward apoptosis. Specifically, the marked downregulation of the anti-apoptotic protein Bcl-2 observed upon Menin inhibition is important, as Bcl-2 overexpression conventionally acts as a terminal safeguard against mitochondrial outer membrane permeabilization (MOMP) and can contribute to intrinsic chemoresistance in cancer [27–29]. Conversely, the concomitant increase in Bax and p-p53 is consistent with activation of a p53-associated mitochondrial apoptotic response after 5-FU-induced stress [27]. This mechanistic insight supports the strategy of targeting chromatin-regulatory complexes to resensitize tumors to chemotherapy.

However, the clinical translation of synergistic drug combinations is often hindered by disparate physicochemical properties, decoupled pharmacokinetic profiles, and suboptimal drug ratios at the tumor site [19]. To address this translational barrier, we rationally engineered HLM@BF as an “inside-out” hierarchical nanoplatform with spatiotemporally programmed release logic, consistent with recent principles in multi-drug cancer nanomedicine and nanomedicine translation [30–32]. Within this context, the significance of HLM@BF lies in integrating these established delivery principles into a formulation-function-mechanism framework tailored to the BMF-219/5-FU combination. The disulfide-bridged mesoporous silica core (M) provides a high-surface-area reservoir for co-loading hydrophilic 5-FU and hydrophobic BMF-219 and enables redox-responsive framework degradation under intracellular glutathione (GSH)-rich conditions [33–35]. The pH-sensitive DOPE/CHEMS lipid layer serves as an acid-responsive gatekeeper to reduce premature leakage and may facilitate intracellular release through endo/lysosomal destabilization after cellular uptake [36–39]. The exterior hyaluronic acid (HA) decoration supports CD44-associated uptake in CD44-expressing gastric cancer cells and may enhance tumor accumulation together with the enhanced permeability and retention (EPR) effect [40–42]. Therefore, the intended distinction of HLM@BF is not a single material component, but the coordinated integration of HA-associated uptake, pH-triggered liposome destabilization, and GSH-triggered core degradation to deliver this hydrophilic/hydrophobic drug pair at a therapeutically relevant ratio.

From a pharmacological perspective, this delivery strategy is particularly tailored for BMF-219. As a covalent Menin inhibitor, BMF-219 may benefit from a “hit-and-run” mechanism, in which a transiently high local concentration potentially leads to rapid target occupancy. HLM@BF was designed to exploit pathological hallmarks of the tumor microenvironment (TME) to approximate this kinetic profile. The sequential protonation-induced lipid destabilization and GSH-triggered silica collapse enable accelerated release of the payloads under intracellularly relevant conditions [43]. Beyond improving therapeutic efficacy, this material-enabled kinetic alignment may also contribute to biosafety. Consistent with the known sensitivity of hepatic safety markers to lipid nanoparticle formulation and liver exposure, the non-HA-decorated LM@BF induced mild hepatic stress potentially related to formulation-dependent hepatic interaction and reticuloendothelial system exposure [44]. In contrast, the exterior HA modification of HLM@BF, together with EPR-associated tumor accumulation [45], was associated with stronger tumor fluorescence, while hepatic stress markers remained less pronounced in this model. By reducing systemic dilution and improving tumor delivery, HLM@BF may help align BMF-219 exposure with 5-FU-induced DNA damage, translating our epigenetic findings into a more tolerable therapeutic strategy.

Despite these encouraging results, several limitations warrant further investigation. First, while the subcutaneous xenograft model effectively demonstrates tumor growth inhibition, it cannot fully recapitulate the orthotopic tumor microenvironment (TME) or the intricate immune landscape of GC. Future studies utilizing patient-derived xenograft (PDX) or orthotopic models are highly warranted [46]. Second, while our study focused on intracellular apoptotic signaling, emerging evidence suggests that the functional restoration of p53 can fundamentally alter the tumor immune microenvironment, overcoming the immune evasion typically driven by p53 suppression or mutations [47–49]. Investigating potential synergy between HLM@BF and immune checkpoint inhibitors (ICIs) may represent a promising avenue for achieving more durable antitumor responses. Third, although the siTP53 perturbation experiment supports the involvement of p53-associated signaling in the HLM@BF response, future work should include stable p53-knockout, p53-mutant, or isogenic rescue GC models to define genotype-specific efficacy more rigorously. Fourth, although HA was introduced through DSPE-PEG-HA within the liposomal layer and supported by physicochemical and uptake-related evidence, absolute HA surface density was not directly quantified. Future work using fluorescently labeled HA, colorimetric HA assays, or other orthogonal surface quantification methods would further refine this parameter. Fifth, ex vivo fluorescence imaging provides quantitative carrier-level biodistribution information, whereas formal drug-level pharmacokinetic profiling of free and encapsulated 5-FU and BMF-219 in plasma and tissues remains an important direction for future translational studies. Dedicated pharmacokinetic, long-term toxicity, and large-animal studies will be required before clinical translation.

In conclusion, through comprehensive in vitro and in vivo investigations using a GC xenograft model, we demonstrated that the HLM@BF nanoplatform enhances antitumor activity while reducing systemic toxicity. The developed HLM@BF nanoplatform enables the co-delivery of a Menin inhibitor and a chemotherapeutic agent through cascade-responsive materials engineering, thereby improving the chemosensitivity of GC cells through Menin- and p53-associated apoptotic signaling. This work provides mechanistic insight into Menin-mediated 5-FU resistance and supports further development of nanotechnology-assisted combinations of epigenetic intervention and cytotoxic therapy.

## Supplementary Information

Supplementary Material 1. Supplementary Figure 1: Ubiquitination assay, bioinformatic screening, high-performance liquid chromatography (HPLC) quantification, optical tracking, and biosafety profiles. A. Immunoprecipitation (IP) and ubiquitination assay evaluating the ubiquitination status of Menin after treatment with dimethyl sulfoxide (DMSO) or BMF-219, with or without the proteasome inhibitor MG-132. Menin was immunoprecipitated and ubiquitinated Menin species were detected by Western blotting using an anti-ubiquitin antibody. Input lysates were used to verify ubiquitin, Menin, and ACTB expression. B. Top 10 fluorouracil-associated interacting genes retrieved from the Comparative Toxicogenomics Database (CTD), highlighting TP53 as a major fluorouracil-associated gene. C. Pearson correlation analysis between TP53 and Men1 expression in gastric cancer based on the Gene Expression Profiling Interactive Analysis 2 (GEPIA2) database. D. Men1 expression levels in normal tissues and gastric cancer tumor tissues from The Cancer Genome Atlas stomach adenocarcinoma cohort (TCGA-STAD) analyzed through UCSC Xena. E-F. Representative HPLC chromatogram of BMF-219 (E) and corresponding standard calibration curve (F). G-H. Representative HPLC chromatogram of 5-FU (G) and corresponding standard calibration curve (H). I. Ultraviolet-visible (UV-Vis) absorption spectra of M, LM, HLM, M@BF, LM@BF, and HLM@BF, confirming Cy5.5 loading and optical tracking capability. J-L. Post-treatment hematological analysis of red blood cell (RBC) count (J), white blood cell (WBC) count (K), and platelet (PLT) count (L) in mice treated with saline, free BF, LM@BF, or HLM@BF. M-P. Serum biochemical analysis of liver function markers, including alanine aminotransferase (ALT) (M) and aspartate aminotransferase (AST) (N), and renal function markers, including urea (UREA) (O) and creatinine (CREA) (P), after different treatments. Data are presented as mean ± standard deviation (SD). ^*^P < 0.05, ^**^P < 0.01, ^***^P < 0.001; ns, not significant.

## Data Availability

The raw data and source data supporting the findings of this study are available from the corresponding author upon reasonable request.

## Abbreviations

5-FU: 5-fluorouracil
BF: BMF-219 and 5-FU
CAFs: Cancer-associated fibroblasts
DL: Drug loading
EE: Encapsulation efficiency
EMT: Epithelial-mesenchymal transition
EPR: Enhanced permeability and retention
GC: Gastric cancer
GSH: Glutathione
HA: Hyaluronic acid
HLM: HA-modified pH-sensitive liposome-coated M
HLM@BF: HA-modified pH-sensitive liposome-coated M@BF
Lipo: pH-sensitive liposomes
LM: pH-sensitive liposome-coated M
LM@BF: pH-sensitive liposome-coated M@BF
M: Disulfide-bridged mesoporous silica nanoparticles
M@BF: BF-loaded M
MDR: Multidrug resistance
PDI: Polydispersity index
qRT-PCR: Quantitative real-time polymerase chain reaction
RES: Reticuloendothelial system
RNA-seq: RNA sequencing
TME: Tumor microenvironment
